# Neutrophil Gelatinase-Associated Lipocalin (NGAL) as a Biomarker of Acute Kidney Injury (AKI) in Dogs with Congestive Heart Failure (CHF) Due to Myxomatous Mitral Valve Disease (MMVD)

**DOI:** 10.3390/ani15111607

**Published:** 2025-05-30

**Authors:** Maria Chiara Sabetti, Sabrina Fasoli, Serena Crosara, Cecilia Quintavalla, Giovanni Romito, Roberta Troìa, Francesca Fidanzio, Chiara Mazzoldi, Erica Monari, Francesco Dondi

**Affiliations:** 1 Department of Veterinary Sciences, University of Parma, Strada del Taglio 10, 43126 Parma, Italy; mariachiara.sabetti@unipr.it (M.C.S.); cecilia.quintavalla@unipr.it (C.Q.); francesca.fidanzio@unipr.it (F.F.); 2 Department of Veterinary Medical Sciences, Alma Mater Studiorum—University of Bologna, Via Tolara di Sopra 50, Ozzano dell’Emilia, 40127 Bologna, Italy; sabrina.fasoli2@unibo.it (S.F.); giovanni.romito2@unibo.it (G.R.); chiara.mazzoldi2@unibo.it (C.M.); erika.monari3@unibo.it (E.M.); f.dondi@unibo.it (F.D.); 3 Section of Small Animal Emergency and Critical Care, Department of Small Animals, Vetsuisse Faculty, University of Zurich, 8057 Zurich, Switzerland; roberta.troia.vet@gmail.com

**Keywords:** acute kidney injury, cardiorenal syndrome, heart failure, renal biomarker, tubular damage

## Abstract

Dogs with acute congestive heart failure (CHF) can develop acute kidney injury (AKI); the prevalence of this condition has not been defined. This study aimed to assess the occurrence of AKI (increase in serum creatinine (sCr) ≥ 0.3 mg/dL) within 48 h from admission in dogs with myxomatous mitral valve disease (MMVD) with acute CHF, and the role of urinary neutrophil gelatinase-associated lipocalin (uNGAL) as a predictive marker of AKI. This was a multicentric, prospective observational study. Thirty dogs were included. The types and dosages of the diuretics administered, as well as the serum and urinary chemistry, including uNGAL and uNGAL, to the urinary creatinine ratio (uNGALC), were determined at admission (T0) and after 24 (T24) and 48 (T48) hours of hospitalization. Nineteen dogs developed AKI. We found no statistically significant differences in sCr, uNGAL, uNGALC, diuretic dosage, or hours of hospitalization between dogs that developed AKI and those that did not. The urinary NGAL and uNGALC values were not statistically significantly different at any time point, while the sCr was higher at T24 and T48 than T0. Our findings suggest that AKI in MMVD dogs with CHF is primarily functional, driven by effective decongestion rather than severe tubular damage, with the benefits of decongestion outweighing transient increases in sCr.

## Simple Summary

The heart and kidneys are strictly related organs, and the failure of one of them could lead to dysfunction of the other. In this study, we aimed to assess the development of in-hospital acute kidney injury in dogs with congestive heart failure due to myxomatous mitral valve disease undergoing emergency treatment. The second aim was to evaluate the role of urinary neutrophil gelatinase associated-lipocalin, a widely studied biomarker of renal tubular damage, as a predictor of acute kidney damage. It was found that the 63% of dogs with congestive heart failure developed kidney injury within the first 48 h of admission. However, the urinary biomarker’s levels were not associated with kidney dysfunction, and it was not coupled with diuretic treatment. These findings suggest that mild worsening renal function during congestive heart failure is common in dogs. However, it appears to be an expected hemodynamic response to successful decongestion and does not reflect renal tubular damage. This study can contribute to the understanding of the interplay between the heart and kidneys in dogs affected by MMVD.

## 1. Introduction

The term cardiorenal syndrome (CRS) describes the connection between the cardiovascular and renal systems, whereby the acute or chronic dysfunction of one of these two organs may lead to the acute or chronic dysfunction of the other [1,2,3,4].

The CRS type 1, or acute cardiorenal syndrome, refers to the development of acute kidney injury (AKI) in the course of acute congestive heart failure (CHF) [3,5]. Irrespective of the different hemodynamic profiles that might affect these patients, both the reduction in effective circulation volume or the increase in central venous pressure can lead to decreases in the glomerular filtration rate and AKI development [3,5,6]. In addition, the non-hemodynamic causes of CRS type 1 include neurohormonal activation, tubular collapse due to increased renal interstitial pressure, chronic inflammation, and oxidative damage [3,5]. In humans, worsening renal function is an important independent prognostic factor for adverse events, including cardiovascular mortality, longer length of in-hospital stays, and CHF recurrence [7]. Although such renal impairment is usually regarded simply as an acute increase in serum creatinine (sCr) ≥ 0.3 mg/dL (https://static1.squarespace.com/static/666b9ecb4064a156963b4162/t/67cb04e9e54f3e17317349f9/1741358315504/4_ldc-revised-grading-of-acute-kidney-injury.pdf (accessed on 26 May 2025)), this definition has some limitations. Notably, sCr has drawbacks in diagnosing and quantifying CRS type I, as its relationship with GFR is non-linear and exponential, particularly in acute settings. Moreover, its elevation is insensitive to detecting AKI and to estimating the extent of tubular damage [1,7,8,9]. For this reason, over time, a multitude of alternative and complementary AKI biomarkers, including neutrophil gelatinase-associated lipocalin (NGAL), have been investigated in human medicine, with controversial results [7,8,10].

The occurrence of renal damage in dogs affected by different cardiac diseases has been described in the veterinary literature [2,11,12,13]. However, in this species, the characterization of this injury has not been consistently documented, especially in the setting of acute CHF, and has traditionally been based primarily on the value of sCr, which might increase as a consequence of appropriate decongestion and an adequate diuretic response [12]. Interestingly, a study including a small sample of healthy dogs and dogs with acute CHF due to myxomatous mitral valve disease (MMVD) found that the latter had higher serum NGAL than the former. Moreover, dogs developing worsening renal function within 7 days showed higher serum NGAL concentrations upon admission compared to those with stable sCr, thereby highlighting the potential role of NGAL as an early biomarker of AKI during acute CHF [14]. These preliminary findings suggested that combining sCr changes with tubular damage biomarkers, such as NGAL, may provide better insights into AKI in canine CHF than relying solely on sCr values.

Given the above, in the current study, we aimed to (1) assess the occurrence and prevalence of AKI in dogs with MMVD with acute CHF, (2) characterize it, and (3) evaluate the role of urinary NGAL (uNGAL) in predicting AKI during acute CHF.

## 2. Materials and Methods

### 2.1. Study Design

This is a multicentric prospective observational study performed at two Veterinary University hospitals (University of Parma and University of Bologna), between April 2020 and May 2021. The study was approved by the local Scientific Ethical Committee for Animal Testing (Protocol N. 747 of 13 October 2016). An informed consent was obtained from the owners of the dog included.

### 2.2. Study Population

Privately owned dogs with a previous or newly diagnosed MMVD, made according to the current guidelines of the American College of Veterinary Internal Medicine (ACVIM) [15], were considered eligible for the present study. To be included in the study, the dogs had to have presented with acute CHF requiring emergency in-hospital treatment with an IV loop diuretic for at least 24 h.

The diagnosis of CHF was based on the following criteria: clinical signs consistent with CHF (e.g., increased respiratory rate and effort, cough, exercise intolerance); radiographic evidence of pulmonary edema including interstitial and/or alveolar lung pattern; and/or the evidence of lung B lines associated with left atrial enlargement at the point-of-care echocardiography [16].

All dogs without a previous echocardiographic diagnosis of MMVD were subjected to a complete echocardiographic exam after the resolution of CHF signs. The echocardiographic examinations were performed by a board-certified cardiologist (G.R. or S.C.) at both centers (Philips Epiq CVx, Bothell, WA, USA). The criteria for the current ACVIM guidelines were used to further classify the dogs (e.g., as affected by MMVD at ACVIM stage C) [15].

Dogs were excluded from the study in the case of having other acquired or congenital cardiac disease, International Renal Interest Society (IRIS) stage 3 and 4 chronic kidney disease (CKD), sepsis, pyuria shown in the fresh urine sediment examination (>5 white blood cells per high power field), urinary tract infection, and received nephrotoxic drugs within the month prior to admission. The presence of atrial fibrillation secondary to MMVD did not preclude inclusion.

Healthy dogs (*n* = 46) and dogs with stable MMVD (*n* = 98) at different ACVIM stages, included in a previous study on uNGAL performed by our research group [13], were used for comparative purposes. The definition of stable dogs refers to “dogs free from clinical and radiographic signs of CHF at the time of enrollement” [13].

### 2.3. Therapeutic Management

At the time of admission for acute CHF, the enrolled dogs were allowed to receive an IV loop diuretic, either furosemide or torasemide, administered through repetitive boluses or a constant rate infusion, at the discretion of the attending clinicians. In addition to loop diuretics, various additional cardiovascular drugs and oxygen supplementation could be administered based on the animal’s medical history and clinical status. Based on the patients’ needs and clinical stability, their non-invasive blood pressure (SunTech^®^ Vet20TM Veterinary Blood Pressure Monitor, SunTech Medical, Inc., Morrisville, NC, USA; petMAP Graphic, Ramsey Medical, Sydney, Australia) and pulse oximetry (Nellcor SpO2 module for Dash 3000, GE Healthcare, Rahway, NJ, USA) measurements were evaluated. Previous treatment with angiotensin-converting enzyme inhibitors (ACEi) was discontinued at the time of hospitalization.

### 2.4. Data Collection

For the purposes of the study, complete clinical and clinicopathological data were collected at the time of hospital admission (T0). as well as after 24 h (T24) and 48 h (T48) of hospital stay.

Specifically, at T0, blood was collected via standard venipuncture using blood vacuum collection systems. Concurrent fresh urine samples were collected via spontaneous voiding or cystocentesis [13] within one hour from hospital admission and diuretic administration. The sampling was repeated at T24 (24 h from T0) and T48 (48 h from T0). The blood was collected in plain tubes with gel separators, allowed to clot at room temperature, and then centrifuged and divided into two aliquots. One aliquot was analyzed within 60 min of collection to assess the parameters necessary for managing the hospitalized patient, while the other was stored at −80 °C for up to 2 months and used for research purposes (sCr, urea, C-reactive protein (CRP), and serum electrolytes). The urine samples were centrifuged (1000× *g* for 10 min) and examined within 1 h of collection. For the uNGAL measurements, aliquots of the urine supernatant were stored at −80 °C for up to 2 months until their analysis.

The following clinicopathological analyses were performed at the clinical pathology laboratory of one of the centers (University of Bologna): serum chemistry, including sCr, urea, CRP, and serum electrolyte measurements (AU480, Beckman Coulter, Brea, CA, USA); urinalysis, including urine specific gravity measurements evaluated using a hand-held refractometer (American Optical, Buffalo, NY, USA), dipstick tests (Combur-Test^®^ 10 UX, Roche, Rotkreuz, Switzerland) read by an automated reader (URISYS 1100, Roche, Switzerland), and the evaluation of urine sediment at low (100×) and high (400×) power fields; urinary chemistry, including urinary creatinine (uCr), total protein concentrations, urine protein-to-creatinine ratio (UPC), and urinary electrolyte measurements (AU480, Beckman Coulter, Brea, CA, USA).

In addition to the aforementioned clinicopathological variables, the urinary NGAL was measured using a commercial sandwich ELISA according to the manufacturer’s instructions (Dog NGAL ELISA kit, BIOPORTO Diagnostics, Hellerup, Denmark) and as previously reported [13]. Aliquots of the urine supernatant samples of dogs with CHF enrolled in the study were stored at −80 °C for up to 2 months until assayed. The assay was performed as previously described [13]. The assay was validated in our laboratory for the dogs following a validation protocol including linearity and intra-assay variations, and the validation results were similar to those previously reported and consistent with those reported by the manufacturer [17]. Specifically, the regression coefficient’s linearity check yielded a calculated R2 of 0.985 (*p* < 0.001) in each run. The intra-assay CV was determined by calculating the standard deviation (SD) and mean of the two duplicate measurements for each dog, and then dividing the SD by the mean, resulting in a CV of 6.3%. Urine samples from the CHF dogs, at an initial dilution of 1:100, were used, followed by1:300, 1:500, and 1:900 dilutions for samples where the analyte concentration could not be determined. The concentration of uNGAL in the samples was determined by measuring the absorbance of the solution at 450 nm using an appropriate plate reader (DV990BV4 spectrophotometer, N.T. Laboratory s.r.l. Calenzano, Italy) and calculated from a standard curve using curve-fitting software (GraphPad Prism software, version 6, San Diego, CA, USA). The results are expressed as the uNGAL concentrations (pg/mL) and uNGAL-to-uCr ratio (uNGALC; pg/mg).

### 2.5. Definition of AKI and Animal Grouping

Acute kidney injury was defined as an increase in sCr of ≥0.3 mg/dL within 48 h from admission and was graded based on IRIS guidelines (https://static1.squarespace.com/static/666b9ecb4064a156963b4162/t/67cb04e9e54f3e17317349f9/1741358315504/4_ldc-revised-grading-of-acute-kidney-injury.pdf (accessed on 26 May 2025)). For a better understanding of the fluctuation of the sCr values over time, in addition to the sCr levels obtained at T0, T24, and T48, baseline sCr values were retrospectively retrieved from patients’ medical records when available. These values were based on sCr measurements taken within one month of the onset of CHF, which led to their inclusion in the study. For the same reason, sCr values obtained after seven days of admission were recorded when available.

For the purpose of our analysis, the enrolled dogs were grouped based on the occurrence of AKI 48 h from their admission into two categories: CHF AKI and CHF no-AKI.

### 2.6. Data Analyzed

In addition to the aforementioned clinicopathological variables, at T0, the following data were collected: signalment, medical history including comorbidities and prior treatments, body weight, physical and radiographic examination findings. At T24 and T48, we collected body weight measurements and CHF treatment characteristics, including the need for and duration (hours) of oxygen supplementation, as well as the type, dose (mg/kg/day), and route of administration of loop diuretics in the first 24 and 48 h of hospitalization.

Additional data included the duration of hospitalization and outcome, defined as survival from hospital discharge or death (with the latter further distinguished into spontaneous death or death due to euthanasia).

### 2.7. Statistical Analysis

The data distribution was assessed graphically and using the Shapiro–Wilk test. The data were expressed by means of standard descriptive statistics and are presented as the mean ± SD or median and range (minimum–maximum) based on the normal or non-normal data distribution, respectively. Variables of interest in the dogs with CHF were compared among different time points using the Wilcoxon test for paired samples and paired samples t-test (T0 vs. T24; T24 vs. T48; T0 vs. T48). The Kruskal–Wallis test with Dunn’s post hoc tests with Bonferroni correction, the Mann–Whitney U test, and Student’s t-test were used to compare different study groups (healthy dogs vs. stable MMVD vs. CHF dogs; CHF AKI vs. CHF no-AKI dogs). For all comparisons (paired time points: T0 vs. T24; T24 vs. T48; T0 vs. T48; pairwise comparisons: CHF AKI vs. CHF no-AKI dogs), differences were reported using mean differences and 95% confidence intervals (for normally distributed variables) or median differences using the Hodges–Lehmann estimator with 95% confidence intervals for non-normally distributed variables. Categorical variables among groups were assessed using both the Chi-squared test and Fisher’s exact test for 2 × 2 tables. The Spearman’s correlation coefficient was used to assess potential correlations between variables. The results were considered significant if *p* < 0.05.

A power analysis was conducted to estimate the minimum required sample size for our study. Based on previously reported data indicating an AKI incidence rate of 48% in dogs with CHF [12], we set the alternative hypothesis accordingly. The null hypothesis assumed an AKI incidence rate of 25%. Using a type I error (α) of 0.05 and type II error (β) of 0.2 (power = 80%), the minimum required sample size was calculated to be 31 dogs. Statistical analyses were performed using commercially available statistical software packages (MedCalc Statistical Software version 19.5.1; Ostend, Belgium and GraphPad Prism version 10.2.2; GraphPad Software, San Diego, CA, USA).

## 3. Results

### 3.1. Baseline Characteristics

The study population consisted of 30 dogs, all at ACVIM stage C; in total, 12/30 (40%) dogs were at their first CHF episode. Overall, 19/30 (63.3%) were males (of which two were neutered) and 11/30 (36.7%) were females (of which three were spayed). Mixed breed dogs (18/30 (60%)) were more common than purebred dogs (12/30 (40%)). The median body weight was 7.7 kg (range, 2.7–29.3 kg), and the mean age was 12 ± 2.2 years. At the time of presentation, the mean respiratory rate was 64 ± 21 breaths/min, and the mean systolic blood pressure was 133 ± 11.6 mmHg. At the time of inclusion, 21/30 dogs were receiving cardiovascular therapy, 15/21 (71%) were receiving oral furosemide (mean dosage 4 ± 1 mg/kg/daily), and 3/21 (14%) were receiving oral torsemide (median dosage 0.4 mg/kg/day, range 0.22–0.5). The mean duration of diuretic therapy before enrollment was 278 ± 195 days. Additional cardiovascular drugs, aimed at controlling MMVD, were associated with loop diuretics, namely pimobendan (21/21 (100%)), an ACE-i (11/21 (52%)), and spironolactone (7/21 (33%)). Moreover, 3/21 (14%) dogs were receiving digoxin for the treatment of atrial fibrillation.

### 3.2. In-Hospital Treatment and Outcome

After admission, all dogs started IV diuretic therapy. Diuretics were administered both in boluses and via constant rate infusion in 21/30 (70%) dogs, whereas 9/30 (30%) were managed exclusively by boluses. Twenty-five dogs received furosemide, whereas 5 dogs received both furosemide and torsemide. For the dogs receiving only furosemide, its mean dosage during hospitalization was 9.8 mg/kg/die. The median dosages for the dogs receiving both furosemide and torsemide were 9.4 mg/kg/die (range 7–11 mg/kg/die) and 0.45 mg/kg/die (range 0.26–0.6 mg/kg/die), respectively.

All but three dogs received oxygen supplementation. The oxygen was administered through a nasal cannula or oxygen cage in 13/27 (48.15%) and 14/27 (51.85%) dogs, respectively. The mean duration of oxygen therapy was 19 ± 7.7 h. None of the included dogs was mechanically ventilated. The enrolled dogs were hospitalized for a median of 49 h (range 24–288 h). At 48 h after admission, 26/30 (86.7%) and 4/30 (13.3%) dogs were alive and dead, respectively; moreover, 11/30 dogs (37%) were discharged between 28 h and 30 h of hospitalization. All deaths were from cardiac-related causes. Among the deceased dogs, 3/4 (75%) subjects died within the first 24 h, whereas 1 dog out of 4 (25%) died after 48 h. Ninety days after discharge, 21/30 dogs (70%) were alive and 9/30 (30%) had died. The mean survival time of the deceased dogs was 23 ± 33 days after enrollment.

### 3.3. Occurrence of AKI in CHF Dogs

Baseline sCr measurements were available in 11/30 dogs (37%), revealing the presence of previous azotemia in 4/11 (36.4%) dogs (sCr median 1.75 mg/dL, range 1.6–2.26). At T0, 6/30 (20%) dogs were azotemic (sCr > 1.6 mg/dL). Nineteen out of 30 dogs (63%) developed AKI within the first 48 h of admission. Specifically, the kidney injury was grade I (non-azotemic) in 10/19 (53%) dogs, grade II in 7/19 (37%) dogs, and grade III in 2/19 (10%) dogs.

The demographic and clinical data for the CHF AKI dogs and CHF non-AKI dogs were comparable. Likewise, the median durations of oxygen therapy and the median doses of furosemide received in the first 24 and 48 h of hospital stay were similar between the CHF AKI dogs and CHF non-AKI ones. No statistically significant differences were detected regarding the durations of hospital stay and outcomes between groups (Table 1).

In the CHF AKI group, 14/19 (73.7%) dogs had available sCr measurements at 7 days. Among these, the sCr concentrations returned to baseline in 10/14 (71.4%) dogs, while 4/14 (28.6%) had persistently elevated concentrations. In the CHF non-AKI group, sCr measurements were available for 7/11 (63.6%) dogs at 7 days, with the sCr concentration remaining stable in 6/7 (85.7%) dogs and increasing (>0.3 mg/dL) in 1 dog out of 7 (14.3%).

### 3.4. Clinicopathological Data Comparison at Different Time Points

The sCr and urea concentrations at T24 and T48 were higher compared to T0 (*p* < 0.001 and *p* = 0.001 for sCr, *p* < 0.001 and *p* = 0.002 for urea, respectively).

The serum creatinine (sCr) and urea concentrations at T24 and T48 were higher compared to T0 (sCr: *p* < 0.01 for both time points; urea: *p* < 0.01 for both time points).

No statistical differences were observed in UPC values over three time points. In addition, the serum chloride concentrations were significantly lower at T24 and T48 compared to the values measured at T0 (*p* = 0.002 and *p* = 0.004, respectively). The serum potassium concentration was statistically significantly lower at T24 compared to T0 (*p* = 0.007). Complete clinicopathological results are reported in Table 2.

### 3.5. Urinary NGAL Evaluations at Different Time Points

The median values for uNGAL and uNGALC are reported in Table 2. Overall, increased uNGAL values above the reference interval were detected in 16/30 (53%) dogs at T0, 16/30 (53%) dogs at T24, and 6/15 (40%) dogs at T48. Similarly, increased uNGALC values were documented in 23/30 (77%) dogs at T0, 22/30 (73%) at T24, and 9/15 (60%) at T48. No significant differences were documented for either uNGAL or uNGALC values among the time points (T0 vs. T24, *p* = 0.51, *p* = 0.72, respectively; T0 vs. T48, *p* = 0.56, *p* = 0.84, respectively; T24 vs. T48, *p* = 0.89 and *p* = 0.90, respectively).

There was no correlation between CRP and uNGAL or uNGALC at T0 (r 0.1, 95% CI: −0.28 to 0.43, *p* = 0.65; r 0.12, 95% CI: −0.25 to 0.46, *p* = 0.51, respectively), T24 (r −0.11, 95% CI: −0.70 to 0.62, *p* = 0.87; r −0.2, 95% CI −0.77 to 0.51, *p* = 0.55, respectively), or T48 (r = 0.14, 95% CI:−0.75 to 0.85, *p* = 0.78; r = 0.08, 95% CI: −0.78 to 0.84 *p* = 0.87, respectively). No correlation was documented between the values for uNGAL and uNGALC and the total dose of furosemide administered during the first 24 h (r = 0.34, 95% CI: −0.09 to 0.61, *p* = 0.09; r = 0.35, 95% CI: −0.02 to 0.65, *p* = 0.08, respectively). Similarly, the values of uNGAL and uNGALC at T48 were not correlated with the total dose of furosemide administered during the first 48 h (r = 0.56, 95% CI: −0.01 to 0.86, *p* = 0.06; r = 0.46, 95% CI: −0.15 to 0.81, *p* = 0.13, respectively). The correlation between uNGAL and the torasemide dosage was not assessed due to the low number of dogs receiving it.

### 3.6. Comparison of Urinary NGAL Between Groups

The values of uNGAL and uNGALC were not significantly different between CHF AKI vs. CHF non-AKI at T0 (*p* = 0.20 and *p* = 0.42, respectively), T24 (*p* = 0.74 and *p* = 0.47, respectively), or T48 (*p* = 0.89 and *p* = 0.89, respectively).

**Figure 1:**
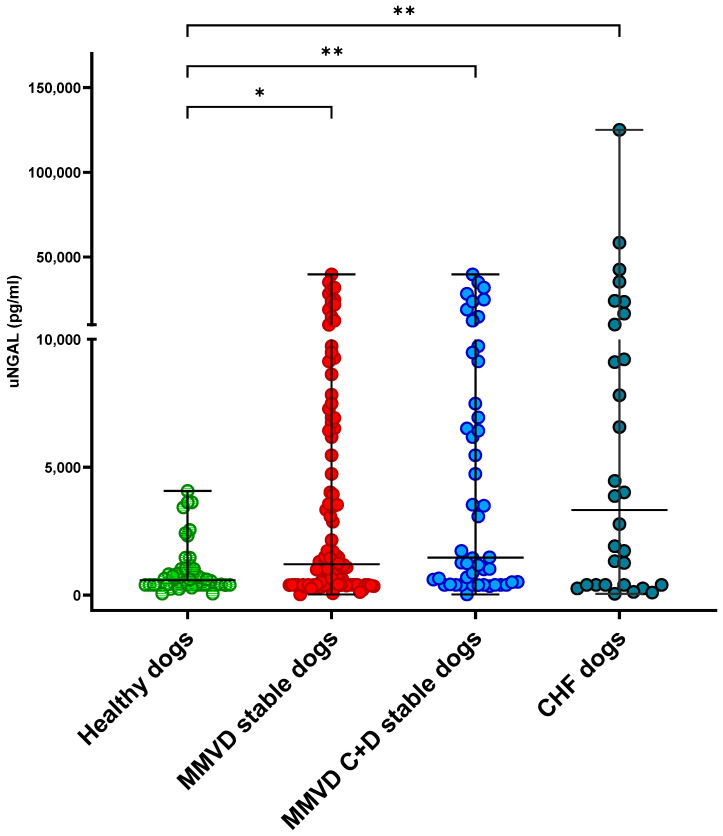
Dot plot showing results of urinary neutrophil gelatinase-associated lipocalin (uNGAL) comparison between healthy dogs (*n* = 46) (green dots) and dogs with myxomatous mitral valve disease (MMVD) (*n* = 98) (red dots), and among dogs with MMVD in ACVIM stages C + D (blue dots) (*n* = 48) and dogs with cardiac heart failure (CHF) (*n* = 30) (grey dots). Upright bars represent minimum and maximum values, while horizontal lines (central bars) represent the median value. Statistical comparisons were performed using a Kruskal–Wallis test followed by Dunn’s post hoc test with Bonferroni correction; * *p* < 0.05, ** *p* < 0.01.

**Figure 2:**
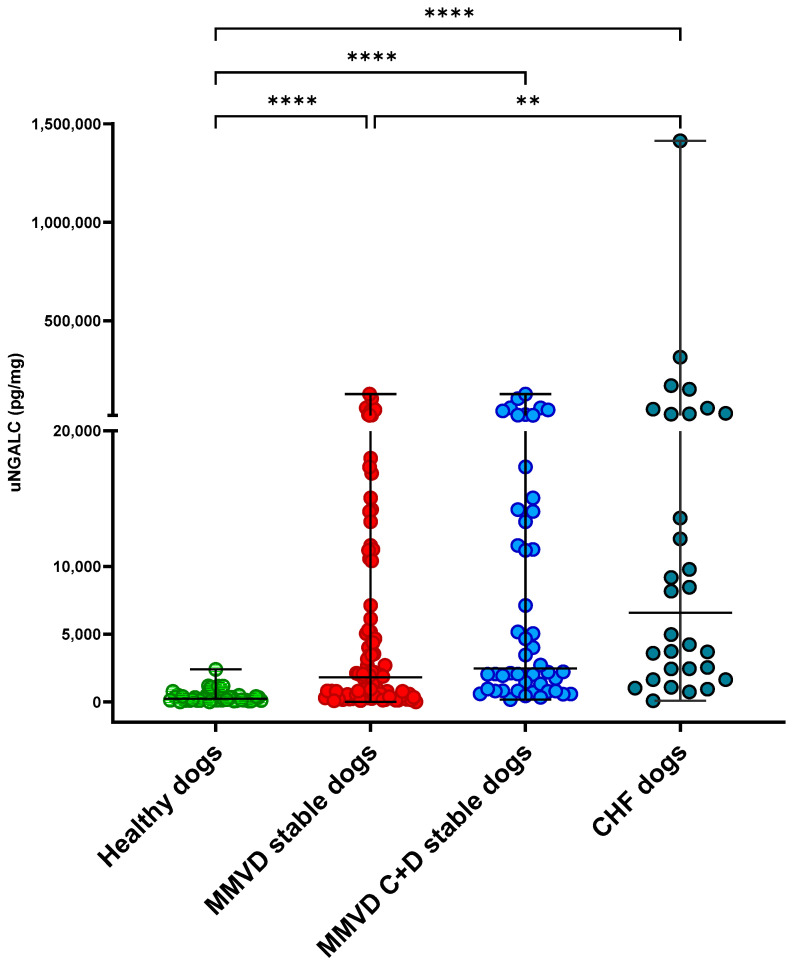
Dot plot showing results of urinary neutrophil gelatinase-associated lipocalin-to-urinary creatinine ratio (uNGALC) comparison between healthy dogs (*n* = 46) (green dots) and dogs with myxomatous mitral valve disease (MMVD) (*n* = 98) (red dots), and among dogs with MMVD in ACVIM stages C + D (blue dots) (*n* = 48), and dogs in cardiac heart failure (CHF) (*n* = 30) (grey dots). Upright bars represent minimum and maximum values, while horizontal lines (central bars) represent median value. Statistical comparisons were performed using Kruskal–Wallis test followed by Dunn’s post hoc test with Bonferroni correction. ** *p* < 0.01, **** *p* < 0.001.

Overall, at T0, the CHF dogs had significantly increased uNGAL (3322 pg/mL; range, 50–124,995) and uNGALC (6578 pg/mg; range, 95–1,413,968) values compared to the healthy dogs (uNGAL, 584 pg/mL; range, 56–4072; uNGALC, 231 pg/mg; range, 15–2407), as well as the stable MMVD dogs (uNGAL 1204 pg/mL; range, 30–39,732; uNGALC 1816 pg/mg; range, 22–127,693) (*p* = 0.002, *p* < 0.001, respectively; Figure 1 and Figure 2). No statistically significant differences were detected when the uNGAL and uNGALC values were compared between the CHF dogs and stable MMVD dogs in stage ACVIM C only (*p* = 0.8 and *p* = 0.1, respectively).

## 4. Discussion

The present study aimed to assess the occurrence and prevalence of AKI during the in-hospital treatment of acute CHF due to MMVD in dogs, and to investigate the diagnostic role of uNGAL in this clinical setting. The main findings of our analysis were as follows: (1) AKI occurred in 63% of dogs with CHF within 48 h of hospitalization; (2) the majority of dogs experienced a normalization of sCr within one week; (3) AKI was not directly linked to diuretic therapy, and did not affect the short-term outcome; (4) despite AKI occurrence, no statistically significant changes in uNGAL values were documented during the study period, with comparable concentrations between CHF AKI and CHF no-AKI.

Acute kidney injury occurring during CHF is referred to as CRS type 1 and represents a well-known entity in humans, with a reported prevalence range of between 25 and 40%. The real epidemiology of such worsening renal function is complex to estimate due to inconsistency in the definition criteria and timeframes considered for its occurrence [18,19,20]. Cardiorenal syndrome type 1 in humans carries a negative prognostic impact, increases healthcare costs, and might predispose sufferers to diuretic resistance [18,20]. On the other hand, changes in sCr induced by diuresis and decongestion may be largely functional or hemodynamic, and consequently clinically benign. Indeed, severe deterioration in renal function is uncommon in people with acute CHF, as most of the events related to worsening renal function involve small- to moderate-sized changes in sCr without elevation of tubular damage biomarkers, representing an expected hemodynamic response to successful decongestion [21].

In contrast, the epidemiology of CRS type 1 in dogs is less well-characterized. The prevalence rates vary among studies due to the variable definitions of renal insufficiency and heterogeneity of cardiac diseases and treatment protocols in the published clinical trials [12,14,22,23]. In a recent retrospective study evaluating dogs with left-sided CHF treated with parenteral furosemide, AKI was reported in 48% of cases, was generally in the non-azotemic range, and was not related to the cumulative in-hospital furosemide dosage or long-term outcome [12]. In the paper by Jung et al. 2018, only 11 out of 31 dogs (35%) with MMVD and CHF developed worsening renal function within 7 days of CHF occurrence; however, dogs experiencing transient azotemia were excluded from the analysis [14]; thus, the percentage of dogs experiencing hyperazotemia after diuretic therapy might be underestimated. In the present study, in-hospital AKI was detected in 19/30 (63%) of CHF dogs; however, all of them remained asymptomatic, and in 70% of them the hyperazotemia was transient.

The pathogenesis of AKI during CHF is complex, with both arterial underfilling and renal venous congestion being involved [3,5,6,7]. Drugs administered during CHF might additionally contribute to worsening renal function. The use of diuretics may resolve congestion and ameliorate global tissue perfusion and function, although over-diuresis could promote kidney hypoperfusion [12,19,20]; drugs working on the renin–angiotensin–aldosterone system, such as ACEi, can further contribute to kidney damage [15,24]. Finally, previous AKI episodes and advanced age can contribute to reducing the renal reserve and the development of CRS type 1 [11,19,24]. In this study, all mentioned factors may have contributed to AKI occurrence in the CHF dogs. Indeed, the majority of the enrolled dogs (60%) had already experienced previous episodes of CHF and could have suffered from previous concurrent AKI episodes. All included dogs were receiving loop diuretics and various cardiac treatments. In addition, many patients (57%) were receiving chronic ACEi therapy before the hospitalization, which was discontinued at the time of admission but might have limited the kidneys’ ability to sustain perfusion in the timeframe before presentation. Ultimately, the study population consisted of elderly patients, with a median age of 12 years, whereas the possibility of aging kidneys, reduced renal functional mass, concurrent CKD, and sub-clinical tubular damage could all coexist, as previously highlighted. However, the age and diuretic dosage did not differ between the dogs in the CHF no-AKI group and CHF AKI group.

Nonetheless, the degrees of worsening renal function documented in most of the cases were mild (mainly AKI IRIS grades I and II), asymptomatic, and transient, and overall did not affect the duration of hospital stay or short-term outcome. Among the four dogs experiencing death, none died because of AKI or AKI-related signs. Thus, the pattern of AKI in our CHF population was apparently benign and more likely reflecting appropriate decongestion. The results concerning uNGAL seem to support the statement mentioned above. Indeed, the uNGAL values did not statistically differ between CHF AKI and CHF no-AKI; moreover, they did not increase during hospitalization. On the contrary, there was a non-significant trend of uNGAL and uNGAL decreasing from T0 to T48. This unexpected result might support the hypothesis that that the mild hyperazotemia experienced in some dogs did not reflect tubular injury but was more probably a clinically benign change in filtration, with no significant impact on tubular cells.

A previous study that investigated the diagnostic potential of serum NGAL in dogs with MMVD and CHF found that serum NGAL, evaluated at the time of hospitalization, was able to predict the occurrence of worsening renal function, defined as a rise in sCr above 1.6 mg/dL within 7 days from decompensation. However, dogs developing transient azotemia that resolved within 24 h were excluded from the analysis [14]. Our study differs because we did not exclude dogs that experienced transient azotemia and we evaluated urinary instead of serum NGAL levels; for these reasons, our results are hard to compare.

Neutrophil gelatinase-associated lipocalin assays have been tested to estimate the risk of AKI in the course of CHF in humans; however, conflicting results have been reported. According to the paper by Palazzuoli and colleagues, admission plasma NGAL was predictive of in-hospital worsening renal function and the post-discharge outcome in a cohort of people with CHF [20]. On the contrary, another study conducted in a similar setting, measuring plasma NGAL levels, revealed no apparent tubular involvement in people with low-grade CHF [8]. Other studies that measured uNGAL reported similar results to ours [21,25,26]. In the work conducted by Ahmad and colleagues, tubular biomarkers, including uNGAL levels, measured at baseline and 72 h, were not related to the development of worsening renal function, which was transient and mild and showed no prognostic implication [21].

It might be hypothesized that the severity of the underlying cardiac condition itself could influence the outcome and development of worsening renal function during treatment. None of the enrolled dogs required non-invasive or mechanical ventilation, nor inotropic support; we cannot rule out that the development of AKI, during more severe forms of CHF, may have a different course and prognostic significance compared to what was documented in our population.

Finally, the long-term effect of temporary worsening renal function, and its impact on diuretic responsiveness and CKD development, are not known. We could not evaluate the prognostic value of uNGAL for the survival time due to the small number of patients and the low mortality rate (13%) documented in this study population.

In the comparison of uNGAL values among healthy dogs and dogs with stable MMVD and MMVD with CHF, the dogs with CHF had higher uNGAL and uNGALC values than the others. However, when only ACVIM stage C stable MMVD dogs were compared with CHF dogs, no statistical difference in uNGAL and uNGALC values was detected. It could be hypothesized that factors such as the severity of cardiac disease and diuretic therapy play a role in the occurrence of some degree of tubular damage. Moreover, these factors may put MMVD dogs with advanced disease at risk for developing more severe kidney disease, regardless of the presence of concurrent CHF. Furthermore, it should be noted that the increases in uNGAL values detected in CHF dogs are significantly lower than those reported in dogs with intrinsic AKI, where the median reported concentrations are 160,095 pg/mg (range: 735–1,241,800) for uNGAL and 202,546 pg/mg (range: 511–3,873,283) for uNGALC [27]. This shows that the AKI observed in our population is primarily functional, due to diuretic treatment, and does not reflect severe tubular damage.

There are several limitations to acknowledge when interpreting the results of this study. The study population was small, and the treatments were not standardized, limiting the value of our results in different critical care settings. In addition, the lack of a basal sCr measurement for the majority of the enrolled dogs was a particular limitation, complicating the renal function evaluation at the time of hospital admission. Moreover, the timeframe chosen to investigate AKI occurrence was limited to the hospitalization period and ranged between 24 and 48 h. Additional tests to characterize tubular damage (e.g., urine protein electrophoresis or other renal biomarkers) could have been helpful in investigating the origin and correlations of urinary NGAL in our study. In addition, although the overall management of enrolled dogs was performed by the attending clinicians in accordance with a board-certified cardiologist (S.C. or G.R.), specific emergency treatments (e.g., the amount and duration of oxygen supplementation, modalities for oxygen supply) were not standardized, for clinical reasons. The majority of enrolled dogs had a mild-to-moderate increase in serum C-reactive protein concentration, which is an expected finding during CHF in dogs [28]. However, we did not perform a methodical screening for additional causes of such an inflammatory state if the clinical and laboratory picture of the dogs did not suggest potential comorbidities. Thus, even in the absence of a correlation between urinary NGAL and serum CRP values based on our results, the inflammatory state of the enrolled dogs was not deeply characterized. Lastly, the sample size was estimated a priori, to detect a difference between a 25% null hypothesis and a 48% expected AKI incidence, as reported in the literature [12]. However, no formal statistical comparison of proportions was performed in the present analysis. As such, an interpretation of negative results should consider the possibility of limited power to detect smaller differences in other study outcomes.

## 5. Conclusions

In conclusion, about two-thirds of the dogs with CHF in this study developed in-hospital AKI, documented by increased sCr; this was mild and transient in most cases and did not affect their short-term survival. The urinary NGAL concentrations were not associated with worsening renal function and did not predict transient AKI in the CHF dogs. These findings support the concept that in the majority of dogs with acute CHF due to MMVD, functional AKI is expected and related to successful decongestion but does not reflect tubular damage, suggesting that the benefits of decongestion may outweigh any modest or transient increases in serum creatinine. The role of recurrent AKI episodes, and their impact on the long-term outcome and diuretic responsiveness, are not known and should be characterized in further studies.

## Figures and Tables

**Table 1:** Population description of two subgroups, namely dogs that developed AKI during hospitalization (AKI) (*n* = 19) and dogs that did not develop AKI during hospitalization (no-AKI) (*n* = 11). Data are presented as the mean ± standard deviation or median (range), depending on the distribution. For each variable, the mean or median difference between groups and the corresponding 95% confidence interval (CI) are reported. Differences were calculated using either the mean difference (for normally distributed variables) or Hodges–Lehmann estimator of median differences (for non-normally distributed variables).

	CHF AKI (*n* = 19)	CHF No-AKI (*n* = 11)	CHF AKI vs. CHF No AKI	*p* Value
			mean/median difference (95% CI)	
Age (years)	12.1 ± 1.8	11.9 ± 2.7	−0.200 (−1.7255 to 1.3255)	0.81
Number of dogs receiving PO furosemide at home	10/19	5/11		0.68
Dosage of furosemide (mg/kg/die)	4 (3–6)	4 (2.5–5.8)	+0.120 (−1.7000 to 1.1700)	0.82
Days of therapy	202 (26–459) (*n* = 9)	321 (24–601) (*n* = 7)	142 (−86 to 399)	0.31
Number of dogs receiving PO torasemide at home	1/19	2/11		
Dosage of torasemide (mg/kg/die)	0.22	0.45 (0.4–0.5)		
Number of dogs receiving PO pimobendan at home	13/19	8/11		
Dosage of pimobendan (mg/kg/die)	0.6 (0.25–0.8)	0.5 (0.25–0.9)	−0.075 (−0.20 to 0.15)	0.66
Number of dogs receiving PO spirolattone at home	3/19	4/11		
Dosage of spirolattone (mg/kg/die)	2.3 (1.5–3)	2 (1.8–2.5)	−0.30	0.72
Number of dogs receiving PO ACEi at home	9/19	2/11		
Dosage of ACEi (mg/kg/die)	0.7 (0.3–1.1)	0.47 (0.25–0.7)	−0.22	0.28
Hours of hospitalization	50 (30–288)	50 (30–120)	0.000 (−12 to 12)	0.72
Total dosage of furosemide administered in the first 24 h of hospitalization (mg/kg)	10.8 (6–16)	12 (7–12.4)	0.000 −2.00 to 1.90	0.81
Total dosage of furosemide administered in the first 48 h of hospitalization (mg/kg)	18 (14–21)	18 (16–19)	−0.5	0.74
Number of dogs alive at 90 days from discharge	15/19	6/11		0.32
Days of survival at 90 days from discharge	4 (2–6) (*n* = 4)	9 (2–71) (*n* = 5)	+5	0.42
Albumin (g/dL) (T0)	2.99 ± 0.31	3 ± 0.54	+0.01 (−0.31 to 0.33)	0.92
Cl (mEq/L) (T0)	109.8 ± 4.9	108.8 ± 4.9	−1.00 (−4.81 to 2.81)	0.64
Creatinine (mg/dL) (T0)	0.92 (0.32–2)	1.18 (0.74–2.14)	0.19 (−0.13 to 0.54)	0.3
CRP (mg/dL) (T0)	3.8 (1–28.9)	3.14 (1.6–20)	−0.26 (−5.27 to 1.40)	0.78
K (mEq/L) (T0)	4.4 (2.9–5)	4.5 (3.4–5.8)	+0.2 (−0.34 to 0.90)	0.34
Na (mEq/L) (T0)	151 (138–156)	149 (147–156)	0.00 (−3.00 to 2.00)	0.89
Total protein (g/dL) (T0)	6.37 ± 0.7	6.37 ± 0.42	0.00 (−0.47 to 0.47)	0.99
UPC (T0)	0.28 (0.08–2.6)	0.31 (0.14–2.7)	+0.06 (−0.09 to 0.86)	0.34
Urea (mg/dL) (T0)	59 (28–200)	59.8 (28–125)	+10 (−22.0 to 61.2)	0.53
uNGAL (pg/mL) (T0)	2774 (50–42,565)	9216 (262–124,994)	+4749 (−1062 to 23,729)	0.20
uNGALC (pg/mL) (T0)	4243 (94–315,514)	25,361 (746–1,413,967)	+8164.96 (−2789 to 2762)	0.42
uNGAL (pg/mL) at 24 h after hospital admission	2915 (400–59,685)	2237 (27–148,765)	+254 (−2515 to +55,475)	0.74
uNGALC (pg/mL) at 24 h after hospital admission	5085 (768–65,372)	3682 (76.4–1,565,945)	1657.42 (−3469 to 153,114)	0.47
uNGAL (pg/mL) at 48 h after hospital admission	1438 (29.4–50,537) (*n* = 11)	1117 (184–51,708) (*n* = 4)	+195	0.89
uNGALC (pg/mL) at 48 h after hospital admission	3600 (225–75,181) (*n* = 11)	2361 (528–193,448) (*n* = 4)	−85	0.89

Abbreviations: AKI: acute kidney injury; CRP, C-reactive protein; uNGAL, urinary neutrophil gelatinase-associated lipocalin; uNGALC, urinary neutrophil gelatinase-associated lipocalin-to-urinary creatinine ratio; UPC, urine protein-to-creatinine ratio.

**Table 2:** Clinicopathological variables of enrolled dogs and study population at the time of admission (T0) and after 24 h (T24) and 48 h (T48). Data are presented as the mean ± standard deviation or median (range), depending on the distribution. For each variable, the mean or median difference between paired time points and the corresponding 95% confidence interval (CI) are reported. Differences were calculated using either the mean difference (for normally distributed variables) or the Hodges–Lehmann estimator of the median difference (for non-normally distributed variables). Abbreviations: CRP, C-reactive protein; uNGAL, urinary neutrophil gelatinase-associated lipocalin; RI, reference interval; uNGALC, urinary neutrophil gelatinase-associated lipocalin-to-urinary creatinine ratio; UPC, urine protein-to-creatinine ratio.

		TIME POINT			Mean/Median Difference (95% CI)			*p* Value		
	RI	T0 *n* = 30	T 24 *n* = 30	T 48 *n* = 15	T0 vs. T24	T0 vs. T48	T24 vs. T48	T0 vs. T24	T0 vs. T48	T24 vs. T48
Albumin (g/dL)	2.75–3.85	2.99 ± 0.4	3.2 ± 0.4	3.2 ± 0.2	+0.2 (0.03 to 0.41)	+0.1 (−0.01 to 0.20)	+0.00 (−0.16 to 0.16)	0.001	0.09	0.33
Chloride (mEq/L)	108.0–118	109.8 (97–117.4)	104.3 (96–122)	97.8 (89–122)	−5.4 (−8.4 to −2.23)	−8.47 (−12 to −4.45)	−3.8 (−8 to −0.35)	0.002	0.004	0.03
Creatinine (mg/dL)	0.75–1.4	0.92 (0.3–2.14)	1.37 (0.88–3.56)	1.4 (0.7–4.5)	+0.39 (0.26 to 0.60)	+0.38 (0.17 to 0.74)	+0.03 (−0.09 to 0.3)	<0.001	0.001	0.61
CRP (mg/dL)	0–1	8.6 ± 9.1	15.3 ± 12.8	11.9 ± 9.5	+4.4 (−0.69 to 9.57)	+1.37 (−6.41 to 9.15)	−3.4 (−2.12 to 5.44)	0.08	0.69	0.22
Potassium (mEq/L)	3.8 –5.0	4.32 ± 0.69	3.9 ± 0.4	3.8± 0.28	0.334 (−0.55 to −0.11)	−0.26 (−0.65 to 0.12)	+0.03 (−0.12 to 0.19)	0.007	0.17	0.80
Sodium (mEq/L)	143–151	149 (138–156)	148 (140–166)	147 (139–155)	−0.5 (−3.0 to 2)	−3 (−5.0 to −1)	−2 (−5.5 to 1)	0.6	0.04	0.11
Total protein (g/dL)	5.6–7.30	6.37 ± 0.6	6.9 ± 0.7	6.7 ± 0.38	+0.55 (0.34 to 0.75)	+0.32 (0.01 to 0.63)	−0.17 (−0.51 to 0.15)	<0.001	0.04	0.27
UPC mg/mg	0–0.5	0.28 (0.08–2.6)	0.34 (0.11–3.6)	0.21 (0.1–2.9)	+0.01 (−0.07 to 0.12)	−0.03 (−0.07 to 0.03)	+0.015 (−0.06 to 0.1)	0.9	0.27	0.72
Urea (mg/dL)	17–48	59 (28–200)	105 (40–235)	101 (49–259)	+35.1 (25.5 to 50)	+42.7 (18.5 to 69.1)	+3.5 (−15.5 to 18.5)	<0.001	0.002	0.84
uNGAL (pg/mL)	0–2300	3322 (50–124.99)	2897 (27–148.76)	1439 (29–51.71)	+506 (2850 to 7679)	−264.06 (−3234 to 2004)	+75.16 (−20,513 to 1039)	0.51	0.56	0.89
uNGALC (pg/mL)	0–1400	6578 (95–1,413,968)	4708 (76–1,565,945)	3601 (225–193,448)	−783.7 (−9088 to 4757)	+356.47 (−2228 to 10,612)	+356 (−2228 to 10,612)	0.72	0.84	0.90

## Data Availability

The data presented in the study are available on request from the corresponding author.

## Abbreviations

The following abbreviations are used in this manuscript:

ACEi	Angiotensin-converting enzyme inhibitor
ACVIM	American College of Veterinary Internal Medicine
AKI	Acute kidney injury
CRS	Cardiorenal syndrome
CHF	Congestive heart failure
CKD	Chronic kidney disease
CRP	C-reactive protein
IRIS	International Renal Interest Society Stage
MMVD	Myxomatous mitral valve disease
NGAL	Neutrophil gelatinase-associated lipocalin
sCr	Serum creatinine
uNGAL	Urinary neutrophil gelatinase-associated lipocalin
uNGALC	Urinary neutrophil gelatinase-associated lipocalin-to-urinary creatinine ratio
uCr	Urinary creatinine
UPC	Urine protein-to-creatine ratio
